# Lack of Detection of XMRV in Seminal Plasma from HIV-1 Infected Men in The Netherlands

**DOI:** 10.1371/journal.pone.0012040

**Published:** 2010-08-10

**Authors:** Marion Cornelissen, Fokla Zorgdrager, Petra Blom, Suzanne Jurriaans, Sjoerd Repping, Elisabeth van Leeuwen, Margreet Bakker, Ben Berkhout, Antoinette C. van der Kuyl

**Affiliations:** 1 Laboratory of Experimental Virology, Department of Medical Microbiology, Center for Infection and Immunity Amsterdam (CINIMA), Academic Medical Center, University of Amsterdam, Amsterdam, The Netherlands; 2 Laboratory of Clinical Virology, Department of Medical Microbiology, Center for Infection and Immunity Amsterdam (CINIMA), Academic Medical Center, University of Amsterdam, Amsterdam, The Netherlands; 3 Center for Reproductive Medicine, Department of Obstetrics and Gynaecology, Academic Medical Center, University of Amsterdam, Amsterdam, The Netherlands; University of California San Francisco, United States of America

## Abstract

**Background:**

Xenotropic murine leukaemia virus-related virus (XMRV) is a recently discovered human gammaretrovirus with yet unknown prevalence and transmission route(s). Its presence in prostate stromal fibroblasts and prostatic secretions suggests that XMRV might be sexually transmitted. We chose to study a compartment closely connected to the prostate, a location where XMRV was detected in independent studies. Seminal plasma samples from HIV-1 infected men were examined as they have an increased probability of acquiring sexually transmitted pathogens.

**Methodology/Principal Findings:**

We studied the prevalence of XMRV in 93 seminal plasma samples of 54 HIV-1 infected men living in The Netherlands with a nested PCR amplification specifically targeting the XMRV gag gene. As a control for the presence and integrity of retrovirus particles, HIV-1 was amplified from the same samples with a PCR amplification targeting the env gene of the virus, or HIV-1 was quantified with a real-time PCR amplifying part of the pol gene.

**Conclusions/Significance:**

Although HIV-1 was amplified from 25% of the seminal plasma samples, no XMRV was detected, suggesting that either the prevalence of XMRV is very low in The Netherlands, or that XMRV is not naturally present in the seminal plasma.

## Introduction

XMRV is the first gammaretrovirus known to infect humans and it has been discovered only recently. Gammaretroviruses are relatively simple retroviruses that are not known to encode accessory genes. XMRV is related to retroviruses from mice and was detected in blood from chronic fatigue syndrome (CFS) patients and healthy controls [1] and in prostate specimens from patients with prostate cancer [2]–[5]. These findings may suggest a transmission route by blood (products) or sexual transmission. The incidence of XMRV infection in patient and control groups varies from 0 to 67%, but has not been investigated systematically in the general population. Higher incidences were often reported from the USA compared to Europe [1]–[11], suggestive of regional differences. A local outbreak of CFS in the USA in 1984 was postulated to be associated with XMRV [1], [8].

XMRV can infect a wide range of cell types and host species in vitro, including human hematopoietic cell lines and prostrate stromal fibroblasts [12]. Furthermore, XMRV is infectious both as a cell–free and as a cell-associated virus [1] and replicates to high titres in prostate carcinoma cell lines [13]. In addition, XMRV has been found in prostatic secretions from men with prostate cancer [14], suggesting the possibility of sexual transmission.

In this study, we have investigated the presence of XMRV and the possibility of sexual transmission of this novel human retrovirus by screening for XMRV in seminal plasma from two groups of HIV-1 infected men, e.g. men having sex with men (MSM, n = 29) and HIV-1 infected heterosexual men from couples seeking fertility treatment (n = 25). HIV-1 infected men were examined in this study as they have an increased probability of acquiring other sexually transmitted pathogens, and because HIV-1 is known to upregulate expression of other retroviruses, especially endogenous retroviruses [15]. As such, they comprise an ideal population to examine the presence of XMRV in a compartment closely connected to the prostate, the only location where XMRV was detected in independent studies, and secondly, to test the hypothesis of sexual XMRV transmission. Furthermore, the detection of HIV-1 can serve as a control for the degradation of retroviral particles in the seminal plasma samples.

## Methods

### Objectives

The objective of this study is to investigate the presence of XMRV nucleic acid in seminal plasma. We hypothesize that XMRV could be present in this compartment, as it has been indisputably found in prostate cancer tissues. We choose to analyse samples from HIV-1 infected men, as they have a higher chance of contracting sexually transmitted pathogens than non-HIV-1 infected men. In addition, the concurrent detection of HIV-1 in the seminal plasma samples can serve as a control for the integrity of retroviral nucleic acid. For 39 patients, a sample from another time-point was available. As virus shedding might not be continuous, those samples were also tested. So, a total of 93 samples from 54 patients were analysed for the presence of XMRV.

### Participants

Seminal plasma was separated from semen collected from either MSM participating in a study examining the semen quality in HIV-1 infected men [16], or from HIV-1 infected heterosexual men undergoing sperm-washing procedures for fertility treatment (for a review of this topic, see [17]). Patients of the latter group were mainly HIV-1 infected by heterosexual contacts, 2 patients were haemophiliacs infected by blood products, and one patient had injected drugs in the past. Patients of the MSM group were not treated with antiretroviral therapy, before or at the sampling moment, while most men in the second group were treated with diverse regimens of antiretroviral drugs. Patient characteristics are summarized in Table 1.

**Table 1:** Characteristics of patients and virus detection results.

	Homosexual men	Heterosexual men	All men
**No. of patients**	29	25	54
**Mean age (range)**	42.4 (32–66)	37.8 (29–46)	40.1
**Origin** *	Dutch: 21Non-Dutch: 2Unknown: 6	Dutch: 18Non-Dutch: 7	Dutch: 39 (72%)Non-Dutch: 9 (17%)Unknown: 6 (11%)
**No. of seminal samples tested**	30	63	93
**Blood plasma HIV-1>50 copies/ml**	28/30	5/63	33/93 (35%)
**Seminal plasma HIV-1>50 copies/ml**	20/30	3/63	23/93 (25%)
**XMRV+**	0/30	0/63	0/93 (0%)

*All men are currently living in The Netherlands.

### Ethics

The MSM were enrolled in a study protocol on the effect of HAART on semen quality. The study was approved by the Institutional Review Board (IRB) of the Academic Medical Centre (AMC) of the University of Amsterdam and all patients gave written informed consent for their semen to be used in general research. Since 2003, the Academic Medical Centre of the University of Amsterdam offers intrauterine insemination (IUI) of HIV-1- serodiscordant couples with a HIV-1 positive man and proven HIV-1 negative woman, and patients are informed that there is no 100% guarantee that infection with HIV-1 will not take place during treatment. All patients sign informed consent.

No specific approval by the IRB of the AMC for the XMRV study was needed in The Netherlands, because the body materials for this study were collected under medical treatment, and can be used for further scientific research when the patients have given consent and samples are analysed anonymously, as was the case here (Code of Conduct as implemented by the Committee on Regulation of Research COREON of the Netherlands Epidemiological Society and the Dutch Federation of Biomedical Scientific Societies that is followed by the IRB of the AMC).

### Nucleic acid isolation

Semen samples were diluted 1∶1 in Hanks balanced salt solution (HBSS; Sigma, St. Louis, MO, USA) and separated by centrifugation at 350 g for 10 minutes. The supernatant ( =  seminal plasma) was stored directly at −80°C, and thawed only once (for this study). Total nucleic acid was extracted from 200 µl of the seminal plasma samples with a method using silica and guanidium thiocyanate [18]. MS2 RNA was added before the extraction, reverse transcribed and subsequently PCR amplified, as an extraction and PCR control. One tenth of the total isolated nucleic acid was reverse transcribed with Superscript III (Invitrogen Corporation, Carlsbad, CA, USA) before performing PCR reactions, such that both viral DNA and RNA will be amplified.

### HIV-1 detection

HIV-1 RNA in seminal plasma was detected with a single PCR amplifying a 723 nt fragment of the env gene. PCR primers for env gene detection were ED31 [19] and 5′ ATGGGAGGGGCATACATTGCT 3′ and PCR reactions were performed with the Superscript™ III One-step RT-PCR System with Platinum® Taq High Fidelity (Invitrogen Corporation Carlsbad, Calif., USA). Amplification was performed for 45 cycles, each cycle involved three steps: 15 sec at 94°C, 30 sec at 55°C, and 1 min at 68°C, plus a final extension of 5 min at 68°C. In addition, seminal plasma samples that were positive for HIV were quantified with a real-time PCR targeting a fragment of the pol gene as described previously [20]. In both assays the limit of detection was 50 copies HIV-1 RNA/ml. HIV-1 blood plasma viral load measurements were done at the Laboratory of Clinical Virology at the AMC (Amsterdam, The Netherlands) with the Versant HIV-1 RNA 3.0 assay (Bayer Diagnostics Division Tarrytown, N.Y.).

### XMRV detection

To detect XMRV sequences in the seminal plasma samples, a reverse-transcriptase (RT) reaction was performed with SuperScript III (Invitrogen, Carlsbad, CA, USA) of 1/10 of the isolated nucleic acid, corresponding to 10 µl of the undiluted seminal plasma sample and the downstream outer primer used in the subsequent first PCR reaction; 5′ GCCGCCTCTTCTTCATTGTTCTC 3′ [1]. A first PCR amplification was performed in a total reaction volume of 50 µL with 1U Taq DNA polymerase (5U/µl, Roche Applied Science, Basel, Switzerland) and the outer primers described by Lombardi et al. [1]: 5′ATCAGTTAACCTACCCGAGTCGGAC 3′ and 5 GCCGCCTCTTCTTCATTGTTCTC 3′, amplifying a fragment of 730 nt of XMRV gag. A second PCR in a total reaction volume of 25 µL containing 5 µL of the first PCR reaction was done with the nested primers 5′GTCTTTAAGTGTTCTCGAGA 3′ and 5′ GGCTTAGGAGGTTTGGACTT 3′, amplifying a fragment of 450 nt of the gag gene of XMRV. Amplification was done for 40 cycles in the first PCR and 30 cycles for the second PCR. Each cycle involved three steps: 1 min at 95°C, 1 min at 55°C, and 2 min at 72°C, plus a final extension of 10 min at 72°C. Samples were tested twice. The detection limit of the XMRV-gag PCR is 5–10 copies input in the PCR reaction, and was assessed with the 730 nt XMRV gag fragment obtained from the 22Rv1 cell line. This fragment was cloned into the TOPO TA vector (Invitrogen, Carlsbad, CA, USA). Gag clones were confirmed by sequencing. Plasmid DNA was isolated, digested with Eco RI and fragments were excised from a 1.5% agarose gel and eluted with the NucleoSpin Extract II system (Macherey-Nagel, Dueren, Germany). Fragment concentration was determined with the Qubit™ Quantitation Platform following the manufacturers' instructions (Invitrogen, Carlsbad, CA, USA); copy numbers were subsequently calculated. As a positive PCR control, we used diluted total nucleic acid extracted from the prostate carcinoma cell line 22Rv1, containing approximately 10 copies of integrated XMRV DNA and producing viral RNA, in each PCR reaction series.

## Results

**Figure 1:**
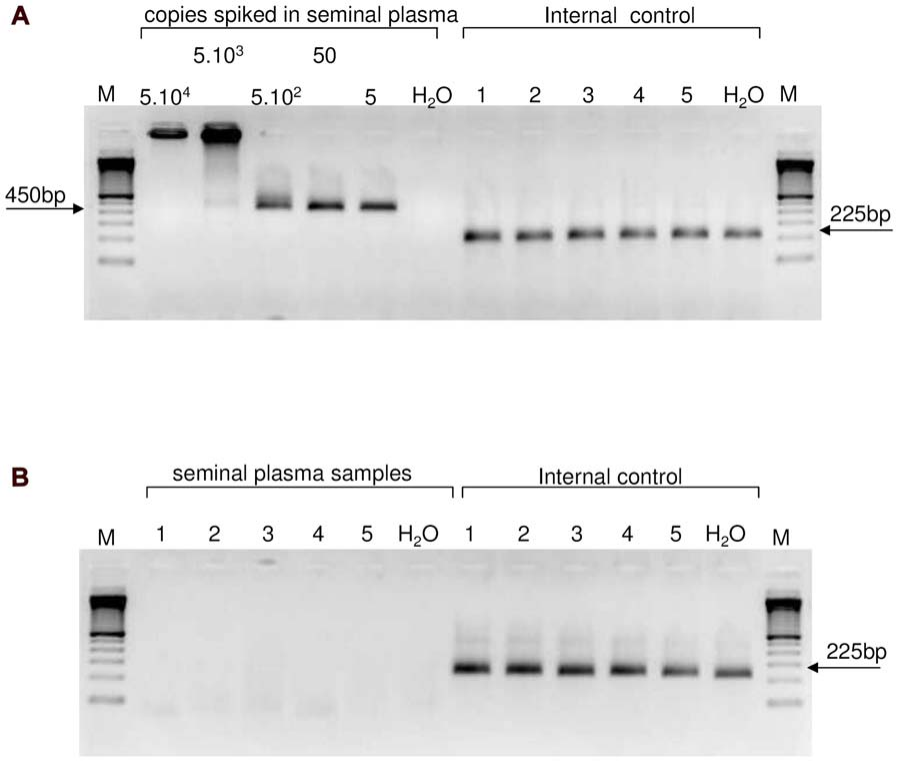
PCR-amplification of XMRV-gag fragments. PCR fragments were analysed on a 2% agarose-gel. (A) Using a cloned XMRV-gag fragment, the sensitivity of our nested PCR reaction amplifying a fragment of 450 bp was determined to be around 5 copies input DNA spiked in XMRV- negative seminal plasma (left panel). High input copy numbers resulted in high molecular weight products that are most likely the result of trapping of excess DNA. MS2 RNA was used as an internal control during the extraction and reverse-transcription reaction, and was amplified in all cases (right panel). (B) PCR-results for five representative patient seminal plasma samples where no XMRV-gag fragments were amplified are shown (left panel). The MS2 internal control fragment of 225 bp was always amplified (right panel), suggesting that no degradation of nucleic acid during extraction, or inhibition during amplification, had occurred.

Ninety-three seminal plasma samples of 54 HIV-1 infected men, 29 infected by homosexual contacts and 25 infected otherwise, but mainly heterosexually, were analysed for the presence of HIV-1 and XMRV. Fig. 1 shows the sensitivity of our nested XMRV gag-PCR that can detect up to 5 copies of an XMRV-gag fragment spiked in seminal plasma (panel A), but did not amplify any XMRV-gag fragments in any patient samples (5 representative patient samples are shown in panel B). In total, in 67% of seminal plasma samples from MSM HIV-1 nucleic acid could be detected, but none of them contained XMRV (Table 1). Five percent of the seminal plasma samples from heterosexual men contained HIV-1, but XMRV nucleic acid was never amplified (Table 1).

In conclusion, no XMRV RNA or DNA was detected in 93 seminal plasma samples of 54 HIV-1 infected men, a group with an increased incidence of sexually transmitted diseases.

## Discussion

The fact that we cannot detect XMRV in the seminal plasma of 54 men, could argue against the presence of XMRV in that compartment and subsequently against sexual transmission of this novel retrovirus, although we can not exclude the possibility that XMRV does not penetrate the seminal plasma. Alternatively, the absence of XMRV could indicate a low prevalence of the virus in Northern Europe. In prostate cancer studies from Germany, the virus was either not detected at all (in 589 tumour samples [6]), or the prevalence was very low: 1/105 samples from prostate cancer patients and in 1/70 samples from men without this condition tested positive for XMRV in another German study [3]. In contrast, in an initial study from the USA, XMRV DNA was found in 8/19 (42%) prostate cancer tissues [2]. In a second North American study 6% of 334 prostate cancer specimens tested XMRV DNA positive, with 23% of these samples expressing XMRV proteins [4]. Also, in a third study, XMRV DNA was found in blood samples from 3.7% of healthy people living in the USA, and in an astonishing 67% of patients with CFS [1]. The latter patients were from a local outbreak of CFS in Incline, Lake Tahoe, USA, with an already suspected viral aetiology. The detection of XMRV in such large numbers could indicate that the virus is spreading in humans and causes local outbreaks [8]. It would be interesting to examine blood and seminal secretions from people living in the Lake Tahoe area for XMRV, although a virus that can infect many people in a short period is less likely to be transmitted only sexually. In line with this, a recent paper reported the detection of XMRV sequences in respiratory secretions, suggesting a possible transmission route by coughing or sneezing [9]. No XMRV was found in (sporadic) CFS patients or healthy controls in three studies from Great Britain and the Netherlands [7], [8], [21], also arguing against a high prevalence of the virus in Europe.

### Limitations

None of the homosexual men studied here were treated with anti-retroviral drugs, but from the heterosexual men, an argument for not detecting XMRV could be that 90% of them are taking antiretroviral therapy, resulting in HIV-1 viral RNA levels in blood plasma below the detection limit. Although protease inhibitors specifically designed to inhibit HIV-encoded protease do not affect XMRV infection and replication, nor do non-nucleoside reverse-transcriptase inhibitors (NNRTI), XMRV is susceptible to the nucleoside reverse-transcriptase inhibitors (NRTI) azidothymidine (AZT) but not tenofovir in one study [22], or to AZT and tenofovir in another study [23]. In our heterosexual patient group, at least 4 patients were taking drug regimens containing AZT, which could obstruct the detection of XMRV. Tenofovir use, however, was not documented. Also, XMRV was found to be susceptible to the integrase inhibitor raltegravir [24]. Raltegravir was approved by the U.S. Food and Drug Administration (FDA) in 2007. As the patients were sampled well before that time, raltegravir use is not an explanation for not detecting XMRV.

A second alternative for not detecting XMRV in seminal plasma could be that the virus is cell-associated, and might be found in semen, but not in seminal plasma. Then, sexual transmission of the virus could still be possible. Or, it could be that XMRV is not excreted at all by the prostate.

A third alternative for the absence of XMRV in our samples could be that the XMRV found in other studies is an inadvertent laboratory contaminant, which has been reported before for other viruses, including another MuLV-related virus [25].

Taken together our results suggest that either XMRV has a low prevalence in this mainly Dutch patient group, as it cannot be found in seminal plasma of 54 HIV-1 infected men, a group with an increased possibility of acquiring sexually transmitted pathogens, or that it is not naturally present in the seminal plasma.
